# Parent-Coached Sibling-Mediated Intervention for Social Communication in Children with Autism Spectrum Disorder: A Pilot Single-Case Study

**DOI:** 10.3390/bs16071240

**Published:** 2026-07-21

**Authors:** Atikah Haira Bagawan, Sarah N. Douglas, Grace Hetke

**Affiliations:** 1 Department of Psychology, Islamic University of Riau, Pekanbaru 28284, Indonesia; 2 Human Development and Family Studies, Michigan State University, East Lansing, MI 48824, USA; sdouglas@msu.edu; 3 Department of Communication Disorders and Sciences, Rush University, Chicago, IL 60612, USA

**Keywords:** autism spectrum disorder, family, intervention, training

## Abstract

This pilot study examined the effects of parent-delivered training and coaching in the *Speak*, *Play* and *Listen* intervention, implemented with neurotypical (NT) siblings to support the social communication development of children with autism spectrum disorder (ASD). Three families, each consisting of a parent, an NT child, and a child with ASD aged 5–12 years, participated in the study. A single-case multiple-probe design was employed to evaluate changes in sibling social interactions. Results showed increases in positive social communication behaviors across all three sibling dyads. Semi-structured interviews indicated that family members perceived the intervention goals, procedures, and outcomes as acceptable, feasible, and socially meaningful. The study highlights the importance of considering family-wide participation, implementation fidelity, and family capacity when designing and delivering interventions for children with ASD.

## 1. Introduction

One of the core challenges that individuals with autism spectrum disorder (ASD) experience is social/communication skills (American Psychiatric Association, 2013). Social/communication skills are essential because they affect how we interact with others in our environment (Fuller & Kaiser, 2020), and in turn impact our ability to establish and maintain relationships with others.

Communication is a dynamic process—it is flexible, depending on the context and the partner, which means that communication development is impacted by the communication opportunities to which children are exposed (Biggs & Meadan, 2018). Children with ASD encounter numerous communication partners daily, such as teachers, peers, and family members, each of whom can elicit opportunities for them to communicate. Yet, successful social/communication development in children with ASD requires communication partners who provide developmentally appropriate learning opportunities in the child’s natural settings (Kent-Walsh et al., 2015). This may include opportunities to respond (Douglas et al., 2021), observational learning (Bandura, 1971), and skills to support their social/communication development (Taylor & DeQuinzio, 2012).

### 1.1. Families in Intervention

Family members are natural communication partners for children with ASD. Numerous studies have explored the effect of parent and sibling-mediated intervention. Findings from a systematic review that analyzed parent-mediated interventions to support social/communication development in children with ASD showed an increase in parent knowledge, parent–child interaction, and child communication (Oono et al., 2013). On top of parents, (Neurotypical) NT siblings of children with ASD also have an important role since sibling relationships are one of the longest-lasting relationships in life. Facilitating positive interactions between siblings can be critical to promoting positive sibling relationships (Douglas et al., 2023). One way to accomplish this goal is to provide NT siblings with communication strategies to support their interactions with their sibling with ASD (Bene & Lapina, 2021). A meta-analysis focused on sibling-mediated intervention and their impact on social communication outcomes for children with ASD found medium effects related to improving social/communication development in children with ASD as a result of sibling-mediated interventions (Bene & Lapina, 2021).

Although parent and sibling-mediated interventions show promise, few have explored the effect of including both parents and siblings in intervention to increase the social/communication development of children with ASD. This gap suggests the need for an intervention framework that targets multiple family relationships simultaneously, rather than focusing on a single family member. Including multiple family members is important as it aligns with family system theory that posits subsystems influence family function (Turnbull et al., 2015). These subsystems include parent–child and sibling relationships. According to this perspective, strengthening one subsystem can produce cascading effects across other subsystems because changes in one family relationship influence interactions throughout the family system. This theoretical perspective informed the design of the current intervention, in which parents were coached to support positive sibling interactions, with the expectation that improvements in sibling social communication would also contribute to broader family functioning. If one subsystem experiences a positive relationship, this consequently affects the other subsystems. When all subsystems have positive relationships, family functioning will increase, which benefits each family member. For example, parents of children with ASD have reported that a challenging sibling relationship can affect the family’s daily functioning (Weiss et al., 2014). One way to increase positive family functioning is to include multiple family members in the intervention in an effort to influence functioning on multiple levels. Therefore, the intervention was designed to engage multiple family members simultaneously in order to strengthen interconnected family relationships and promote positive changes at both the individual and family-system levels.

Interventions that consider family goals, priorities, and abilities, while providing support and resources (Dunst & Trivette, 2009) can build family capacity. Building family capacity is required by law (IDEIA, 2004) and fundamental in early childhood to maximize the child’s learning opportunities. Interventions that increase family skills and knowledge, such as those that involve multiple family members, can build family capacity.

### 1.2. Intervention Implementation Within the Family Context

Implementing intervention in the child’s natural setting with their natural communication partners is essential to supporting generalization. Teaching family members to implement intervention in the home, using materials that the child with ASD is familiar with, and activities that are embedded in their daily routine (e.g., Douglas et al., 2021) promote carryover into the child’s daily life. This can also support family capacity and increase learning opportunities for the child with ASD. Interventions carried out in this manner have also been shown to increase parent empowerment and self-efficacy (Douglas et al., 2022).

Research has explored the impact of training parents to provide instruction and opportunities for their NT children (Douglas et al., 2022; Sheikh et al., 2019). Positive results in target behavior (i.e., the use of modeling AAC to increase communication; play behavior) were noted when mothers supported their NT children as they implemented the targeted strategies (Douglas et al., 2022; Sheikh et al., 2019). Findings indicate that NT siblings displayed more prosocial behavior when mothers provided open and supportive communication (Orm et al., 2022). Similarly, positive results have been noted from training parents to listen and validate NT siblings’ understanding of their sibling’s disability, their emotions, as well as how they perceive challenges in the family (Haukeland et al., 2020). Improvement in parent–sibling communication, knowledge about their sibling’s disorder, adaptation, and emotional and behavioral challenges were all noted (Haukeland et al., 2020). Given these findings in the literature, it may be important to teach parents active listening and open communication strategies to be later used when NT siblings are involved in interventions to ensure that they are reinforcing appropriate sibling interaction.

Sibling-mediated interventions often focus on teaching NT siblings social/communication skills to reinforce sibling interaction (Shivers & Plavnick, 2015), with the *Stay–Play–Talk* intervention being one of most studied interventions (Kryzak & Jones, 2017; Tsao & Odom, 2006; Tsao, 2020). The *Stay–Play–Talk* intervention encourages NT sibling to *Stay* with their sibling as they *Play* and to *Talk* to their sibling about their activity. Sibling-mediated intervention studies that taught NT sibling the *Stay–Play–Talk* intervention consistently showed an increase in positive social behaviors that include communication initiation and response in NT siblings, and the same result has been noted in the sibling with disabilities (Tsao & Odom, 2006; Tsao, 2020). These results provide evidence of observational learning by the child with disability and the benefit of modeling the target behavior by the NT siblings. However, while *Stay–Play–Talk* helps create more opportunities for siblings to interact, it offers less guidance on how NT siblings can keep the interaction going when their sibling with ASD has difficulty initiating, responding to, or maintaining communication. As a result, NT siblings may benefit from simple, evidence-based strategies that help them better support meaningful reciprocal interactions during play. Therefore, the authors developed *Speak*, *Play*, *and Listen*, a modified version of the *Stay–Play–Talk* intervention that adds these communication strategies while keeping the original play-based approach.

### 1.3. Speak, Play and Listen Intervention

The development of *Speak*, *Play and Listen* has been published previously. This intervention encourages NT siblings to use three strategies that have been shown to have positive effects for both siblings. The first step, *Speak*, teaches NT siblings the different ways they can provide communication opportunities to their sibling with ASD. This includes asking questions, providing choices, and commenting. The second step, *Play*, teaches NT siblings social skill strategies such as sharing, organizing play, and compromising. The final step, *Listen*, teaches NT siblings to wait for at least 5 s after communicating to give their sibling time to process and communicate, and allows the NT sibling to attend to their sibling’s communication.

*Speak* is included due to the findings from sibling-mediated interventions that suggest teaching NT siblings to model and provide communication opportunities can promote reciprocal interaction between siblings. After including *Speak* as the first component, *Play* is another component that is included within *Speak*, *Play* and *Listen* because it is a developmentally appropriate activity for both siblings and can be highly motivating. The use of highly motivating activities is important to ensure that siblings have a positive experience (Ferraioli et al., 2012), and play activities often serve this purpose. Additionally, various social/communication skills are embedded within play activities, such as imitation, joint attention, social initiations, and reciprocal communication (Bruinsma & Gengoux, 2020). These opportunities can support social/communication development in children with ASD; hence, it is not surprising that most existing sibling-mediated intervention studies targeted these skills (Banda, 2015).

To wait and *listen* to the sibling’s response is a strategy that is often missing in the sibling-mediated intervention literature, including in the *Stay–Play–Talk* intervention. Including ‘wait’ as a component in an intervention has been evaluated in studies that work with children with complex communication needs, highlighting the importance of waiting for at least 5 s (Sun et al., 2023; Yorke et al., 2018). One study evaluated NT sibling’s use of *Plan*, *Talk*, *Wait*, and *Respond* had found an increase in communication for the sibling with complex communication needs (Douglas et al., 2018). Another intervention used for natural communication partners of children with complex communication needs is aided language modeling, where waiting for at least three seconds before providing another communication opportunity is suggested (Douglas et al., 2022). Although evidence for the listen/wait strategy has primarily been reported in interventions for children with complex communication needs, similar principles have also been demonstrated in ASD research. Providing children with ASD sufficient time to process communication opportunities and formulate responses has been shown to increase response opportunities and appropriate responding (Lamella & Tincani, 2012). Therefore, the *Listen* component of *Speak*, *Play* and *Listen* adapts this evidence to sibling-mediated play by encouraging NT siblings to pause, observe, and allow their sibling with ASD adequate time to initiate or respond during interactions.

Unlike traditional parent-mediated interventions, this study used a cascading training model in which parents coached the neurotypical sibling, rather than directly implementing the intervention themselves. This approach aimed to build the capacity of multiple family members while supporting natural sibling interactions in everyday routines. (see Table 1). This model has been found to yield positive results and build family capacity (Douglas et al., 2022). Due to the involvement of multiple family members in this intervention and various barriers that can hinder family participation in the intervention (i.e., rural area, cost, and time-restraints), there is a need to utilize delivery models that will ensure accessibility. Telepractice has been found to be a feasible and effective delivery model for providing intervention to families of children with ASD and has received high satisfaction from the parents (Akemoğlu et al., 2022; Pacia et al., 2021). Studies that have utilized telepractice to train caregivers in evidence-based practices have been found to be effective in developing caregiver skills (Cox & Davis, 2019).

The feasibility and validity of the *Speak*, *Play and Listen* intervention was obtained from stakeholders (i.e., parents and NT siblings) in a previous study (Bagawan et al., in press). Participants in this study indicated the components that they liked and provided suggestions for adjustments to the intervention. These suggestions were used to inform the intervention used in this study.

This study builds on prior research and addresses important gaps within the literature. The aim is to explore the effect of parent-delivered training and coaching of the *Speak*, *Play* and *Listen* intervention via telepractice. Research questions in the study include the primary research question: Is there a functional relation between parent-delivered training/coaching and the NT sibling’s rate of positive social behavior per minute? Three secondary research questions were also posed: (a) Is there a functional relation between the parent-delivered training/coaching and the parent’s coaching fidelity?; (b) Is there an increase in positive social behavior of child with ASD with their NT sibling as a result of parent-delivered training/coaching?; and, (c) How do family members perceive the goals, procedures, and outcomes of the parent-delivered training and coaching for NT siblings?

## 2. Method

### 2.1. Measures

Various measures were used to understand the characteristics of the children with ASD, the sibling relationship, and family functioning. A *Vineland* parent interview (Vineland-3; Sparrow et al., 2016) was used to understand the communication and social skills of children with ASD. The *Sibling Relationship Questionnaire* (SRQ; Furman & Buhrmester, 1985) was used to gather information about how parents and NT siblings perceive the relationship between the NT sibling and child with ASD. The measure explores levels of conflict, warmth, power, and rivalry between the two siblings. The *Child–Parent Relationship Scale* (CPRS; Pianta, 1992) was used to explore parent perceptions of their relationship with the NT sibling and their conflict, closeness, and dependence. The *Family Communication Scale* (FCS; Olson & Barnes, 2004) gathered information about family communication in the home. The *Family Satisfaction Scale* (FSS; Olson, 2010) determined how satisfied parents were with their family functioning.

These standardized measures were included to characterize child communication profiles, sibling relationships, and broader family functioning before and after the intervention. They were not intended to serve as primary indicators of intervention effectiveness; rather, they provided descriptive context for interpreting sibling interaction outcomes, parent coaching fidelity, and family perceptions of feasibility and acceptability.

### 2.2. Participants and Settings

Families were recruited through flyers posted on various social media groups, including ASD organizations and parent support groups for children with ASD. Families were eligible to participate if they had a (a) parent/legal guardian; (b) child diagnosed with ASD age 5–12 years; and (c) NT child age 8–13 years old, each of whom were willing to participate. The study was conducted in the participants’ home using the toys/materials available in the home to ensure a naturalistic setting. Family members who met the inclusion criteria and were interested in participating provided informed consent/assent and completed various pre-study forms/activities. Parents completed a demographic form for themselves and their children through Qualtrics, a Vineland parent interview; the *Sibling Relationship Questionnaire* (SRQ), a pre-study interview; *the Child–Parent Relationship* Scale (CPRS); the *Family Communication Scale* (FCS); and *the Family Satisfaction* Scale (FSS). NT siblings completed a child version of the SRQ and a pre-study interview. After the study was completed, the parent, NT sibling, and child with ASD completed a post-study interview. The parent also completed the CRPS, FCS, and FSS. At the beginning of the study, four families were recruited to participate (see Table 2). However, the last family withdrew after the initial intervention sessions because the NT sibling was consistently demonstrating the targeted interaction strategies during natural play, leaving limited opportunity for further skill acquisition. Prior to enrollment, families completed a pre-study interview; however, a direct assessment of the sibling’s proficiency with the specific intervention strategies was not conducted, as these strategies were intentionally withheld before the intervention to minimize bias.

#### 2.2.1. Family 1

The first family included Hellen, a 45-year-old female with an associate degree and mom to three children. Hellen participated with her daughter Sara and her son Sam. Sara was the firstborn daughter who was NT, 10 years old, and in fourth grade. Sam was the third and youngest child who was six years old and had ASD. Sam received ABA, speech, and occupational therapy and was in first grade. Sam’s Adaptive Behavior Composite (ABC) on *Vineland-3* was 62 and below the normative mean with relative weakness in communication and socialization domains. Hellen reported that Sara and Sam interacted infrequently. Through the SRQ, both Hellen and Sara reported no power difference between Sara and Sam, as well as a moderate level of warmth/closeness, low levels of conflict, but high rivalry.

#### 2.2.2. Family 2

The second family included Yvonne, a 37-year-old female who was a dentist and mom to three children. Yvonne participated with her sons Darien and Josh. Darien was the second born son who was NT, 10 years old, and in fourth grade. Josh was a first-born child who was 12 years old and had ASD. Josh received ABA therapy and was in sixth grade at the time of the study. On the *Vineland-3*, Josh’s ABC of 44 was below the normative mean with relative weakness in communication, daily living skills, and socialization domains. Yvonne reported that the sibling pair interacted frequently in the past, but their interaction became harder the past two years due to Josh’s challenging behavior and Darien maturing. Through the SRQ, Yvonne reported that Josh had some more power than Darien; whereas, Darien reported that Josh had a lot more power towards him. Both Yvonne and Darien reported that Darien and Josh had high levels of warmth/closeness, low levels of conflict, and a moderate level of rivalry.

#### 2.2.3. Family 3

The third family included Kari, a 43-year-old female who was a professor and had three children. Kari participated with her daughters, Emma, and Raina. Emma was the second born who was NT, seven years old, and in second grade. Raina was the first daughter, was 10 years old, and had ASD and ADHD. Raina received occupational therapy and was in fourth grade.

On the *Vineland-3*, Raina’s ABC of 70 was below the normative mean with relative weakness in socialization. Kari reported that Emma and Raina’s interactions were sometimes positive, but Emma often became frustrated with Raina. Through the SRQ, both Kari and Emma reported that Raina had some level of power towards Emma, that they had moderate levels of warmth/closeness, and a moderate level of sibling rivalry. Kari reported no conflict between the sibling pair; whereas, Emma reported low levels of conflict.

### 2.3. Research Design

A single-subject multiple probe design (Ledford & Gast, 2018) was used to evaluate whether there is a functional relation between parent-delivered training and coaching and the rate of positive social behavior for NT siblings and children with ASD. Data were coded using Datavyu version 1.3.8, a software that supports video data coding (Datavyu Team, 2014). Data were graphed and visually analyzed to determine the trend, level, stability, immediacy of effect, and overlap between baseline and training (Kratochwill et al., 2010).

### 2.4. Materials

Materials included parent training, sibling training, and materials within the home used during at-home activities. Parent training was provided via zoom using power point slides, audio explanation, and discussion between the researcher and parent participant. Content of the parent training included a rationale for the intervention, materials to facilitate training with their NT children, and instruction related to coaching. Training for NT children included power point slides with live examples and discussion between the child and their parent. Each participant had internet and a computer or tablet they used during training, as well as materials within the home (e.g., art supplies, puzzles, kinetic sand, modeling clay, action figures, blocks) that were used during sessions.

### 2.5. Procedures

The study was conducted in two phases: baseline and coaching. In baseline parents used either an iPad or their smart phone that was connected to Zoom with both children and the activity in view of the camera. Children were only instructed to play as they normally played and no further instructions were given. The observer turned off the camera and audio during the session and recorded the play interaction between siblings for 5–10 min. The range of 5–10 min was kept so the NT siblings do not feel pressured to keep the activities going if the siblings were not comfortable to do so. During data collection, the researcher used the speaker view to ensure that the children were focused on the screen.

After baseline, parents and the NT siblings received training. Parent training was administered via Zoom, facilitated by the first author, and took approximately 45–60 min (see Table 1). Parents were provided with the foundation and rationale of the intervention, the training materials for siblings, and how to provide coaching for siblings. The training for the NT sibling was delivered by the parent and took approximately 30 min. Training started with a discussion of diversity, the NT sibling’s characteristics, characteristics of the child with ASD, general information about ASD characteristics, types of communication, the *Speak*, *Play* and *Listen* strategies, problem-solving, and emotional support for the NT sibling. At the end of training the parent developed an action plan with the NT sibling for use in their first session.

Following training, NT siblings went through coaching that was carried out by the parent. The first author recorded all coaching sessions via Zoom with audio and video off. Coaching included: (a) pre-observation discussion; (b) sibling dyad interaction during a play activity; and (c) post-observation discussion. In pre-observation, the parent discussed the sibling’s use of *Speak*, *Play* and *Listen* since training, reviewed the strategy, planned which materials were needed, and discussed the kind of talk that they could engage in with their sibling with ASD. Then the parent observed the siblings play for 5–10 min. During post-observation, the parent had the NT sibling reflect on their interaction, what went well, and what they would do differently next time. Then the parent provided supportive feedback followed by corrective feedback and thanked the NT sibling for their efforts. Lastly, the first author asked parents if they had any questions, concerns, or if there were any issues that needed to be addressed. In the first coaching session, the first author provided feedback to the parent on how well they delivered the coaching to their NT child, and what needed to be covered in the next session. Coaching continued until mastery was reached, which was defined as three sessions above the highest data point in baseline on the NT sibling’s rate of positive social behavior.

#### 2.5.1. Procedural Fidelity

Procedural fidelity was assessed separately for baseline, parent training, NT sibling training, and parent coaching using phase-specific checklists. Baseline fidelity included five items: pressing record on Zoom, keeping both children in camera view, indicating when the session started, recording 5–10 min of interaction, and avoiding instruction during the session. Parent training fidelity was based on completion of the 56 planned steps covering the intervention rationale, sibling training materials, and coaching procedures. NT sibling training and parent coaching were scored with 23-step and 18-step checklists, respectively. Because no a priori threshold was set for acceptable fidelity, fidelity data are reported descriptively and interpreted as feasibility and internal-validity information.

#### 2.5.2. Dependent Measure

The primary outcome in the study was the rate of NT siblings’ positive social behavior (see Table 3). Positive social behavior included both positive social initiations and positive social responses. Negative social behavior included negative social initiations and negative social responses. This was also coded but were not included as a primary dependent variable. The rate of positive social behavior per minute by the child with ASD was also measured as a secondary variable. Parent’s coaching fidelity was measured to ensure high fidelity of coaching implementation to NT siblings. This consisted of three parts (i.e., pre-observation discussion, observation of sibling interaction during a play activity, and post-observation discussion) and was measured as a percentage.

#### 2.5.3. Inter-Observer Agreement

Research assistants were trained until they were able to code behaviors of interest to 90% accuracy. Coder training included review of operational definitions, practice coding with sample videos not included in the final dataset, comparison with master-coded examples, and discussion until coders reached at least 90% agreement before formal coding. Disagreements during IOA checks were reviewed after independent coding was completed and were resolved through consensus for reporting purposes.

Interobserver agreement (IOA) was calculated point by point by taking the number of agreements divided by the number agreements plus disagreements and multiplying by 100. Any disagreements between the coders were discussed until resolved. Videos were randomly selected across phases and participants and were completed by research assistants who are blind to the study purposes and phases. IOA was completed for 40% of sessions in baseline for all sibling dyads, 40% of coaching sessions for Sara–Sam, and Emma–Raina, and 50% of coaching sessions for Darien–Josh.

IOA for the primary dependent variable (i.e., NT sibling’s rate of positive social behavior) in baseline was 80% for Sara (range = 75–100%), 100% for Darien, and 83% for Emma (range = 83–100%). IOA in training phase was 84% for Sara (range = 81–87%), 93% for Darien (range = 89–97%), and 95% for Emma (range = 89–100%). For the secondary dependent variable (i.e., child with ASD’s rate of positive social behavior), IOA in baseline was 100% for Sam, 100% for Josh, and 88% for Raina (range = 86–89%). IOA in training phase was 100% for Sam, 93% for Josh (range = 94–100%), and 97% for Raina (range = 94–100%).

#### 2.5.4. Social Validity

Social validity was measured before and after intervention via interviews. Parents, NT siblings, and children with ASD engaged in a pre-study interview with the first author, and post-study interview with a research team member to avoid bias in responses. Questions for pre- and post-study interviews were designed to understand how participants perceive the goals, procedures, and outcomes of the intervention.

Interviews were semi-structured and designed to elicit participant perspectives on intervention goals, procedures, perceived outcomes, barriers, and suggestions for improvement. Responses were reviewed descriptively to identify recurring topics across participants rather than analyzed using a formal qualitative methodology; therefore, social validity findings are presented as descriptive feasibility evidence rather than as evidence of intervention effectiveness. For children with ASD, interview questions were simplified and yes/no response options were used when appropriate to support communication accessibility.

## 3. Results

**Figure 1:**
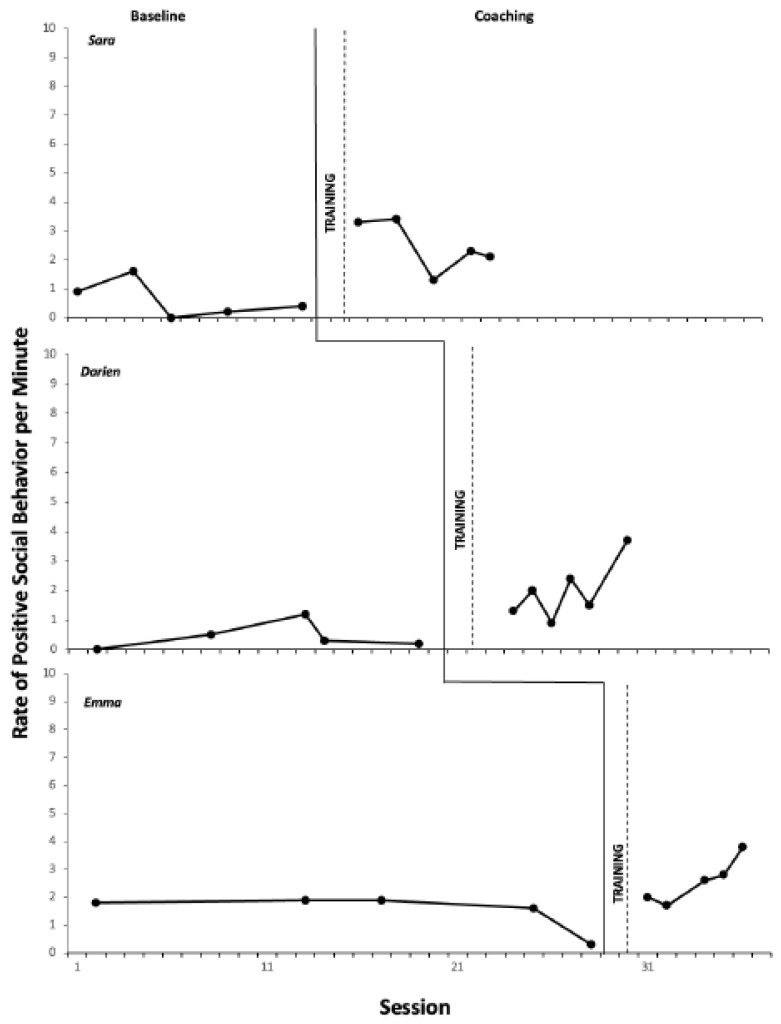
NT sibling positive social behavior.

**Figure 2:**
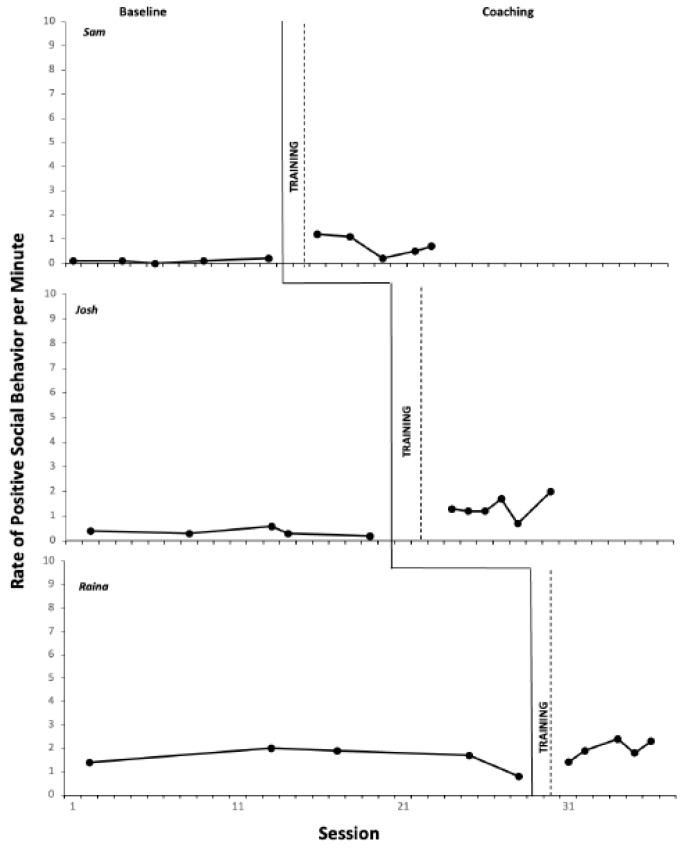
Child with ASD positive social behavior.

The data suggested support for a functional relation between the parent-delivered *Speak*, *Play* and *Listen* intervention and increases in positive social behavior for NT siblings (see Figure 1) and siblings with ASD (see Figure 2). This interpretation was based on changes in level and trend after the staggered introduction of the intervention and at least three intervention data points above the highest baseline point for the NT sibling outcome. However, several dyads showed overlap between baseline and intervention phases, and the size and consistency of change varied across participants.

Parent coaching fidelity was generally strongest during the first few coaching sessions though it declined in later sessions for some families (see Table 4). Social validity results indicated that family members generally found the goals, procedures, and outcomes of the intervention acceptable and meaningful, while also identifying areas where additional support may be needed.

### 3.1. NT Sibling Rate of Positive Social Behavior

#### 3.1.1. Sara

At baseline, Sara’s overall mean rate of positive social behavior was 0.62 per minute (range = 0–1.6) with a descending trend and limited variability. Immediate effect was noted once she entered coaching and continued with limited variability with the third session overlapping with baseline. Sara’s overall mean rate of positive social behavior in intervention increased to 2.48 per minute (range = 1.3–3.4).

#### 3.1.2. Darien

At baseline, Darien’s overall mean rate of positive behavior was 0.44 per minute (range = 0–1.2) with a stable trend and limited variability. His first and third coaching sessions overlapped with baseline data, while higher rates were observed during the second, fourth, and sixth coaching sessions. Darien’s overall mean rate of positive social behavior in coaching increased to 1.96 per minute (range = 0.9–3.7), although the coaching data showed variability.

#### 3.1.3. Emma

At baseline, Emma’s overall mean rate of positive behavior was 1.5 per minute (range = 0.3–1.9) with a descending trend and little variability. Higher rates were observed after the third coaching session, and her overall mean rate of positive behavior increased to 2.58 per minute (range = 1.7–3.8). Emma’s coaching data showed an ascending trend with limited variability.

### 3.2. Sibling with ASD Rate of Positive Social Behavior

#### 3.2.1. Sam

At baseline, Sam’s overall mean rate of positive social behavior was 0.1 per minute (range = 0–0.2) with a stable trend and no variability. Sam’s positive social behavior increased as Sara received coaching with limited variability. Although his third session overlapped with baseline, his positive social behavior had an ascending trend until the last coaching session. Following Sara’s increase in positive social behavior in coaching, Sam’s positive social behavior increased to 0.74 per minute (range = 0.2–1.2).

#### 3.2.2. Josh

Josh’s overall mean rate of positive behavior at baseline was 0.36 (range = 0.2–0.6) with a stable trend and limited variability. There was a notable increase overall mean rate of positive behavior of 1.36 per minute (range = 0.7–2) with some variability for Josh when Darien entered coaching. Although there was overlap on the fifth coaching session with his baseline data, Josh’s positive social behavior showed a stable trend when Darien was in coaching.

#### 3.2.3. Raina

At baseline, Raina’s overall mean rate of positive behavior was 1.56 per minute (range = 0.8–2) with a descending trend and little variability. Raina’s first, second, and fourth coaching sessions overlapped with baseline data, while higher rates were observed during Emma’s third and fifth coaching sessions. Raina’s overall mean rate of positive behavior increased to 1.96 per minute (range = 1.4–2.4).

### 3.3. Parent Coaching Fidelity

Parent coaching fidelity differed across families. Hellen averaged 55.5% fidelity (range = 44.4–77.7%), Yvonne averaged 65.7% (range = 50–83.3%), and Kari averaged 77.72% (range = 33.3–94.4%). Fidelity was often stronger in earlier sessions and declined in later sessions for Hellen and Kari. Across families, parents consistently reminded NT siblings of the *Speak*, *Play* and *Listen* strategies and supported reflection or feedback, but they more often omitted discussion of strategy use since training, opportunities for NT siblings to ask questions, and post-observation space to express concerns. This pattern suggests that parents may have prioritized more directive coaching components, which has implications for simplifying the protocol in future applications.

### 3.4. Social Validity

Social validity interviews provided descriptive insight into how parents and siblings experienced the intervention. Because responses were not analyzed using formal qualitative coding, the quotes below are used to illustrate participant perceptions rather than to establish intervention effectiveness. Overall, parents described the procedures as straightforward and useful for both themselves and their NT child. Kari shared “I appreciated that it [was] relatively straightforward and easy to set everything up. Thanks for making simple…I liked it.” Parents also appreciated being included in the intervention because it allowed them to teach their NT child and remind them how their sibling with ASD communicates. Hellen expressed, “I liked [that I get] to teach Sara how to listen and how to wait for [Sam’s] response.”

Despite these noted benefits, parents indicated a preference for the intervention to be done in person with the presence of an expert to overcome challenges such as child motivation and to provide support for coaching, setting up activities, and examples when things were not easily understood. Hellen said that “if there is in person, it will be more easy…the person [can] show [me] and [my] kids examples.” Yvonne shared that “Darien was so excited at the beginning but then just loses momentum” and felt and the in-person component would help with that. Yvonne also expressed more challenges because Josh was going through puberty and presented more challenging behaviors that discouraged Darien.

Parents expressed their satisfaction with the outcomes of the intervention, specifically in sibling interaction, noting that the intervention helped siblings have more reciprocal interactions, learn how to compromise, and do more things together. Kari said that “Raina recognized her role in it as well, and that [the interactions] really became more reciprocal.” Another positive outcome that parents noticed was their NT child learned how to interact better with their child with ASD (i.e., how to get the sibling’s attention, wait), and noted that the NT child generalized the use of strategies after the study. Yvonne shared that “Darien knows how to interact now…we saw Josh responding to Darien…Darien [is] a bit more patient with his response, and if it does not work Darien [will] try something else.”

NT siblings expressed that the procedure of the intervention was “easy to learn” (Darien) and liked that their mother was able to support their learning. They all found listening and responding to their sibling’s communication to be the most helpful strategies in their interaction. NT siblings also shared the outcome that they experienced. Emma shared that sometimes she played with materials differently than Raina and saw that as a barrier in their interaction. On the other hand, Sara shared that “Sam kind of got better with responding [to me]” and Darien shared that “I speak to him [more].” All NT siblings shared that they would recommend for other families to do the intervention. The second also interviewed children with ASD about their experiences. Through a yes/no question, most children with ASD expressed that they liked playing with their NT siblings. Raina also shared that she found that the being in the program “was fun” because she “got to talk and play [together with Emma]”.

## 4. Discussion

This pilot study offers preliminary insight into the implementation of a whole-family intervention designed to support social/communication development in children with ASD. Mean rates of positive social behavior increased for all children with ASD, which is consistent with the idea that communication partners can support interaction by offering choices, asking questions, commenting, responding to multiple communication modes, and waiting for a response (Douglas & Gerde, 2019). However, several sibling dyads showed overlap between baseline and intervention phases, and low rates of positive social behavior occurred during some intervention sessions. These patterns suggest that participant performance may have been influenced by factors such as NT sibling engagement, coaching fidelity, activity selection, child motivation, or family routines. Therefore, the findings should be interpreted as preliminary evidence of feasibility and potential promise.

The practical significance of these changes is also important to consider. In everyday family life, even small increases in positive initiations, responses, waiting, and shared play may help siblings stay engaged with one another for longer periods and create more natural opportunities for communication. Parent and sibling comments suggested that some families noticed more patience, reciprocity, and attempts to continue interaction during play. However, because the study did not include maintenance, generalization, or direct measures of broader sibling relationship quality, it is not yet clear whether these observed behavior changes translated into lasting improvements in everyday sibling interactions.

Although positive social behavior increased for all sibling pairs and social validity responses were generally favorable, parent ratings on the CPRS, FCS, and FSS remained relatively stable from pre- to post-study (see Table 5). This may indicate a couple of things. First, it is likely that personal factors influence the ratings in these scales, such as parent’s current perception of how satisfied they are with their family dynamic, rather than the satisfaction related to the impact of the intervention. Second, during the timespan of the study, there may be maturation effects that impact family functioning and how parents perceive these changes. With the current study design, some families had longer time commitments than others, which means that as they were participating in the study, they were also facing transitional life changes within their families. This finding may also highlight the need to have an additional support for the family, the use of other study design that puts less demand on families, and modification to the intervention itself. It is also important to note that these broad family measures may not be sensitive enough to capture short-term changes from a brief intervention focused mainly on sibling play. They also suggest that observed changes in sibling interaction should not be interpreted as sole evidence of measurable improvement in overall family functioning.

For Sara and Darien, some of the clearest increases occurred during sessions in which Hellen and Yvonne had higher pre-session coaching fidelity. This pattern is relevant and aligns with prior work showing the importance of high-fidelity coaching (Douglas et al., 2022; Schieltz et al., 2018). However, this relationship should be interpreted cautiously because, in this study, fidelity and child outcomes were not analyzed in a way that supports causal conclusions. Future studies should examine whether specific coaching components are associated with changes in sibling behavior and whether certain components are more essential than others.

Across parents, several coaching steps were omitted more often than others, especially asking about strategy use since training, inviting the NT child to ask questions before play, and providing space for concerns after the observation. Declining fidelity may have reduced the consistency and quality of support that NT siblings received across later sessions, which could help explain some of the variability in participant outcomes. Parents may have skipped reflective or question-based steps because they required more time, flexibility, and child engagement during brief home routines; whereas, reminders and direct feedback were easier to deliver. Future versions of the protocol may benefit from a shorter checklist, visual prompts for parents, booster sessions, and clearer guidance on which coaching steps are essential. For tele-practice delivery, this type of simplification may be especially important because parents are managing the technology, the activity, and the sibling coaching at the same time.

Characteristics of the child with ASD appeared to influence how families experienced and implemented the intervention. For example, Yvonne reported that Josh was experiencing new aggressive behaviors that the family was addressing with other professionals, and both parents and NT siblings described restricted interests and changing activity preferences as challenges during play. These factors are best understood as implementation considerations rather than direct outcomes of the study. They may help explain variability in sibling engagement and highlight the need for strategies such as offering choices, adjusting activities, and supporting motivation during tele-practice sessions (Minjarez & Bruinsma, 2020).

### 4.1. Limitations and Future Research Directions

Like other studies of this nature, this research had several limitations. Most importantly, the study included only three families, and several participants showed overlap between baseline and intervention phases. Parents also noted that some NT siblings were excited at the beginning but lost momentum over time, which may have influenced both observed behavior and social validity responses. In addition, possible history or maturation effects, lack of maintenance and generalization probes, and the absence of effect size or nonoverlap indices further limit interpretation. Future studies should address these issues through different research design with larger and more diverse samples.

Parents also reported difficulty identifying activities that both siblings could enjoy, largely because of differences in interests and motivation. Future studies may benefit from screening sibling interactions before enrollment, selecting dyads with enough opportunity for skill growth, and providing families with a broader list of activity options. The suggestion to include a third person with expertise in ASD came from participant feedback about needing more support with examples, motivation, activity setup, and coaching feedback; therefore, this should be viewed as a possible refinement to the cascading model.

This study offers a practical example of how a main caregiver can train and coach an NT child during sibling-mediated intervention. At the same time, parents indicated that they wanted more immediate guidance on selecting activities, responding when the child with ASD lost interest, adjusting prompts during play, and maintaining NT sibling motivation across repeated sessions. Future research should build this type of feedback more directly into the coaching process.

### 4.2. Implications for Practice

Including siblings in interventions for children with ASD remains important because siblings are natural play partners who share daily routines, home activities, and community experiences with the child (Glugatch & Machalicek, 2021; Tsao & Odom, 2006). In this pilot study, the main practical contribution is the use of parents as coaches within a telepractice-delivered cascading model. As a study with small samples, these practice implications should be viewed as early and tentative until the model is replicated with larger and more diverse samples.

Involving parents may help embed sibling-mediated strategies into everyday home routines, but the current findings also suggest that families may need practical support to use the model consistently. Future applications may benefit from shorter coaching checklists, structured activity options, visual reminders, and periodic expert feedback focused on activity selection, child motivation, and sibling engagement. These supports would also make the model more realistic for families who are using it without an interventionist physically present in the home.

### 4.3. Conclusions

Overall, this pilot study suggests that a parent-coached, sibling-mediated telepractice model is feasible for some families and may support sibling social communication during play. As a pilot study, the findings are encouraging. Future research with different research design, larger and more diverse families, and more implementation supports for parents are needed to build family capacity and create more natural communication opportunities at home for children with ASD.

## Figures and Tables

**Table 1:** Components of the parent training.

Component	Content & Objective	Training Components, Delivery, Time
Foundation and Rationale	Social/communication skills in children with ASD Including family members in interventions to improve social/communication skills in children with ASD Training and coaching family members Special considerations for including NT siblings (possible outcomes, NT sibling’s abilities/ characteristics, characteristics of sibling with ASD, sibling’s opinion/motivation)	Power point slides with video explanation and discussion between researcher and parent 25 min
Training Materials for Siblings	Everybody is unique and has different characteristics NT sibling characteristics Characteristics of the child with ASD Information about general ASD characteristics Types of communication Introduction to the Speak, Play, & Listen intervention Action plan with NT sibling Problem-solving content Emotional support for NT sibling (i.e., self-regulation)	Power point slides with video examples and discussion between parent and NT sibling 15 min
Providing Coaching for Siblings	Pre-session: Develop action plan/goals with NT sibling Sibling Session: Parent observes siblings Post-session: Parent empowers and provides supportive feedback to NT sibling Encouragement for parent	Power point slides with video explanation and discussion between researcher and parent 10 min

**Table 2:** Participant demographic.

Family	Parent	Parent Age	NT Child	NT Child Age	Child with ASD	Child with ASD Age	Ethnicity
1	Hellen	45	Sara	10	Sam	6	Asian
2	Yvonne	37	Darien	10	Josh	12	Asian
3	Kari	43	Emma	7	Raina	10	White

**Table 3:** Behavioral definition and example.

Behavioral Coding	Definition	Example
Positive Social Initiation	A motor, verbal, and/or nonverbal behavior from the focal child towards a sibling by engaging in a prosocial behavior to evoke a response. Code after 2 s of paused interaction or a topic/activity change.	Greetings, shoulder tap, calling sibling’s name, asking questions, commenting, giving choices
Positive Social Response	A reply that is within prosocial behavior from the focal child within 2 s of an initiation or a response from the sibling.	Nodding, reciprocate play, physical imitation
Negative Social Initiation	A motor, verbal, and/or nonverbal behavior from the focal child towards a sibling by engaging in a challenging/unwanted behavior to evoke a response. Code after 2 s of paused interaction or a topic/activity change.	Hitting, pushing, throwing toys across the room, spitting, mocking
Negative Social Response	A reply that is within challenging/unwanted behavior from the focal child within 2 s of an initiation or a response from the sibling.	Hitting, pushing, throwing toys across the room, spitting, mocking

**Table 4:** Parent coaching fidelity.

Family	Parent	Coaching Session					
		1	2	3	4	5	6
1	Hellen	77.7%	55.5%	55.5%	44.4%	44.4%	-
2	Yvonne	72.2%	83.3%	66.6%	50%	61.1%	61.1%
3	Kari	83.3%	94.4%	88.8%	88.8%	33.3%	-

**Table 5:** Pre- and post-study normative assessment.

Family (Parent, NT Child, Child with ASD)	Child-Parent Relationship Scale (CPRS)		Family Communication Scale (FCS)		Family Satisfaction Scale (FSS)	
	Pre-Study	Post-Study	Pre-Study	Post-Study	Pre-Study	Post-Study
1 (Hellen, Sara, Sam)	Conflict: 1.5	Conflict: 1.5	41	38	Moderate (39)	Moderate (37)
	Closeness: 4.4	Closeness: 3.9				
	Dependence: 2.2	Dependence: 2.7				
2 (Yvonne, Darien, Josh)	Conflict: 3.3	Conflict: 3.9	33	28	Very low (26)	Very low (22)
	Closeness: 3.9	Closeness: 3.4				
	Dependence: 3.5	Dependence: 4				
3 (Kari, Emma, Raina)	Conflict: 2.2	Conflict: 1.6	32	36	Very low (29)	Very low (29)
	Closeness: 4.4	Closeness: 4.8				
	Dependence: 3	Dependence: 2.5				

Note. For CPRS the scale is from 1-5 (not really applies-definitely applies) with a higher score indicating higher level of conflict/closeness/dependence; For FCS the range of scores is from 10–50 (strongly disagree-strongly agree for positive family communication) with a higher score indicating higher level of positive family communication; For FSS the range of scores is from 10–50 (family members are very dissatisfied and are concerned about their family-family members are very satisfied and really enjoy most aspects of their family).

## Data Availability

The data supporting the findings of this study are not publicly available due to privacy and ethical restrictions related to participant confidentiality.
